# DKK1 Induced by 1,25D3 Is Required for the Mineralization of Osteoblasts

**DOI:** 10.3390/cells9010236

**Published:** 2020-01-17

**Authors:** Sungsin Jo, Subin Yoon, So Young Lee, So Yeon Kim, Hyosun Park, Jinil Han, Sung Hoon Choi, Joong-Soo Han, Jae-Hyuk Yang, Tae-Hwan Kim

**Affiliations:** 1 Hanyang University Institute for Rheumatology Research, Seoul 04763, Korea; joejo0517@gmail.com (S.J.); ysbin148@naver.com (S.Y.); rlath109@naver.com (S.Y.K.); hyosun1988@naver.com (H.P.); 2 Department of Translational Medicine, Graduate School of Biomedical Science and Engineering, Hanyang University, Seoul 04763, Korea; 3 Department of Biomedical Sciences, Graduate School of Biomedical Science and Engineering, Hanyang University, Seoul 04763, Korea; soyoung130@hanyang.ac.kr (S.Y.L.); jshan@hanyang.ac.kr (J.-S.H.); 4 Gencurix, Inc., Seoul 08394, Korea; jinil@gencurix.com; 5 Department of Orthopaedic Surgery, Hanyang University Seoul Hospital, Seoul 04763, Korea; spineshchoi@gmail.com; 6 Biomedical Research Institute and Department of Biochemistry and Molecular Biology, College of Medicine, Hanyang University, Seoul 04763, Korea; 7 Department of Orthopaedic Surgery, Hanyang University Guri Hospital, Gyeonggi-do 11923, Korea; jaekorea@hotmail.com; 8 Department of Rheumatology, Hanyang University Hospital for Rheumatic Diseases, Seoul 04763, Korea

**Keywords:** 1,25D3, C/EBPβ, DKK1, osteoblasts, differentiation, mineralization

## Abstract

1α,25-dihydroxyvitamin D3 (1,25D3), the most popular drug for osteoporosis treatment, drives osteoblast differentiation and bone mineralization. Wnt/β-catenin signaling is involved in commitment and differentiation of osteoblasts, but the role of the Dickkopf-related protein 1 (DKK1), a Wnt antagonist, in osteoblasts remains unknown. Here, we demonstrate the molecular mechanism of DKK1 induction by 1,25D3 and its physiological role during osteoblast differentiation. 1,25D3 markedly promoted the expression of both CCAAT/enhancer binding protein beta (C/EBPβ) and DKK1 at day 7 during osteoblast differentiation. Interestingly, mRNA and protein levels of C/EBPβ and DKK1 in osteoblasts were elevated by 1,25D3. We also found that C/EBPβ, in response to 1,25D3, directly binds to the human DKK1 promoter. Knockdown of C/EBPβ downregulated the expression of DKK1 in osteoblasts, which was partially reversed by 1,25D3. In contrast, overexpression of C/EBPβ upregulated DKK1 expression in osteoblasts, which was enhanced by 1,25D3. Furthermore, 1,25D3 treatment in osteoblasts stimulated secretion of DKK1 protein within the endoplasmic reticulum to extracellular. Intriguingly, blocking DKK1 attenuated calcified nodule formation in mineralized osteoblasts, but not ALP activity or collagen synthesis. Taken together, these observations suggest that 1,25D3 promotes the mineralization of osteoblasts through activation of DKK1 followed by an increase of C/EBPβ.

## 1. Introduction

Vitamin D deficiency is characterized by bone loss, increased risk of fractures, and bone mineralization defects, which may lead to bone-related diseases such as osteoporosis, osteopenia, and osteomalacia [1,2,3,4]. Moreover, genetic variants of vitamin D3-related genes are associated with abnormal bone turnover and metabolism of bone diseases [5,6,7,8]. Vitamin D3 supplements are essential treatments for the prevention of bone loss, which underlies the mechanisms for decreasing bone resorption by osteoclasts and increasing bone formation by osteoblasts [9]. Thus, vitamin D3 is a nutrient required for bone health and maintenance.

1α,25-Dihydroxyvitamin D3 (1,25D3) is the most active metabolite of vitamin D3 and plays a major role in the enhancement of bone formation [10]. 1,25D3 exerts its actions via binding with and modulating the vitamin D receptor (VDR), a transcriptional regulator [11,12,13]. 1,25D3 also activates Wnt/β-catenin to facilitate osteoblast differentiation [14,15,16]. Conversely, 1,25D3 induces Dickkopf-1 (DKK1) transcripts to suppress Wnt/β-catenin in bladder and colon cancers [17,18], suggesting that 1,25D3 has tissue-specific effects on Wnt/β-catenin signaling. [19,20,21].

Osteoblast differentiation occurs through a sequential route (proliferation, matrix maturation, and mineralization) regulated by different transcription factors and signaling proteins [22]. In particular, Wnt/β-catenin signaling is currently considered the master regulator of osteogenesis and bone formation [23]. β-catenin, a pivotal transcriptional factor in the ‘canonical’ Wnt signaling pathway, stabilizes in the cytoplasm and translocates to the nucleus, leading to transcriptional activation of target genes involved in osteogenesis and osteoblast differentiation. In addition, the secreted Wnt antagonists sclerostin and DKK1 block Wnt signaling by binding to lipoprotein receptor-related protein 5 (LRP5) and LRP6 receptors. Emerging data suggest that dysregulation of Wnt signaling contributes to bone diseases, such as osteosarcoma, Paget’s disease, osteomalacia, osteogenesis imperfecta, osteoporosis, and ankylosing spondylitis [24,25].

The crucial roles of CCAAT/enhancer binding protein beta (C/EBPβ) in commitment, proliferation, and differentiation of osteoblasts and bone formation have been described [26,27,28]. Previous reports have shown that deletion of the C/EBPβ gene impairs skeleton generation in vivo and that overexpression of C/EBPβ promotes transcription of osteocalcin (OCN), a bone formation marker, and osteoblast differentiation in vitro [27,29,30]. Accumulating evidence suggests that C/EBPβ is tightly regulated during osteoblast differentiation. Furthermore, 1,25D3 stimulates VDR to induce C/EBPβ gene transcription and enhances osteoblast differentiation [11]. To study the role of C/EBPβ in osteoblasts, we previously reported that 1,25D3 transiently induces receptor activator of nuclear factor kappa-B ligand (RANKL) in osteoblasts through activating C/EBPβ [31]. Although DKK1 expression in osteoblasts has been reported [32,33], the relationship between DKK1 and 1,25D3-inducible C/EBPβ, as well as the functional role of DKK1 in osteoblasts, remains unclear.

In this study, we investigate a molecular mechanism of 1,25D3-induced DKK1 expression in osteoblasts and the physiological role of DKK1 in osteoblast differentiation.

## 2. Materials and Methods

### 2.1. Ethics Statement

This study was approved by the Institutional Review Board of Hanyang University Hospital (Seoul: 2017-05-003 and Guri: 2018-07-024). Human knee tissues were collected at Hanyang University Guri Hospital. All subjects provided informed consent in accordance with the Declaration of Helsinki. Twenty-two patients with osteoarthritis (OA, 19 females and 3 males; mean ages 74 ± 7.4 years) who had knee surgery, were enrolled.

### 2.2. Isolation of Primary Osteoprogenitors, Osteoblasts, Osteoblast Differentiation

Soft tissues within surgical bone were removed and minced finely with scissors, followed by washes with 1× PBS containing antibiotics to completely remove all marrow cells and small tissues attached to the bone chips. Bone chips were cultured in growth medium to isolate osteoprogenitors using outgrowth methods [34,35]. Isolated osteoprogenitors obtained from passage 2 to 5 were used in all subsequent cell experiments. Primary osteoprogenitors were expanded and re-plated as for osteoblast differentiation. All primary osteoprogenitors were expanded in growth medium containing Nanomycopulitine (L-X16-100, Biowest, Riverside, MO, USA) checked for mycoplasma using a PCR-based method (6601, Takara, Kusatsu, Shiga, Japan). Once cells reached about 90% confluency, growth media were replaced with differentiation media containing osteogenic inductive agents. Osteoprogenitors were incubated with osteoinductive agents (50 μM ascorbic acid (AA), 10 mM β-glycerolphosphate, and 100 nM dexamethasone) for osteoblast differentiation, as described [36,37]. Differentiation media were changed every 3 days. Osteoprogenitors were incubated with AA for 3 days to differentiate into osteoblasts. At indicated days, early and late stages of differentiation were assessed by Alkaline phosphatase (ALP) activity (K412, Biovision, San Francisco, CA, USA) and staining (85L2, Sigma, St. Louis, MO, USA) and by Alizarin Red (ARS; A5533, Sigma, St. Louis, MO, USA) and Hydroxyapatite (HA; PA-1503, Lonza, Basel, Basel-stadt, Swiss) staining, respectively.

### 2.3. Microarray Data

Microarray data were previously reported [36], and differentially expressed genes were screened by Wnt/β-catenin signaling and its related molecules were reanalyzed and presented in this study. All microarray analyses and visualization were conducted using R.3.4.1.

### 2.4. In Vitro Cell Line

Primary osteoprogenitors/osteoblasts and SaOS2 osteosarcoma cell line (a generous gift from Dr. Heekyoung Chung of Hanyang University, Korea) were maintained in DMEM (L0103-500, Biowest, Riverside, MO, USA) and RPMI1640 (L0498-500, Biowest, Riverside, MO, USA) supplemented with 10% FBS and 1% penicillin/streptomycin, respectively. All cells were cultivated at 37 °C and 5% CO_2_ in humidified conditions.

### 2.5. Constructs, Transfection, and Reagents

Human ALP, bone sialoprotein (BSP), osteoblast-specific element (OSE), and OCN promoters were previously described [31]. C/EBPβ and empty vector, and the human DKK1 promoter were kindly given by Dr. Yun Jong Lee (Division of Rheumatology, Department of Internal Medicine, Seoul National University Bundang Hospital) and Dr. Jeong-Yeon Lee (Department of Medicine, College of Medicine, Hanyang University), respectively [31,32,33]. DKK1 cDNA plasmid (HG10170-CY) and empty vector (CV013) were purchased from Sino biological (Wayne, PA, USA). Recombinant DKK1 protein (120-30) was purchased from Peprotech (Rocky Hill, NJ, USA). Small interfering RNA (siRNA) oligos were purchased from Genolution Inc. (Seoul, Korea). Human DKK1 antibody (AF1096) as DKK1 blockade was purchased from R&D system (Minneapolis, MN, USA). Transfection of primary osteoblasts was carried out using Lipofectamine 3000 (L3000008, Thermo Fisher, Waltham, MA, USA) according to the manufacturer’s protocol. The constructs were transformed by Dh5a (RH617, Dongin Company, Seoul, Korea) and plasmid purification was executed by Nucleobond Xtra Maxi (740424, MACHEREY-NAGEL, Duren, North Rhine-Westphalia, Germany). 1α, 25-Dihydroxyvitamin D3 (D1530, Sigma, St. Louis, MO, USA) was diluted with absolute ethanol and used for further study. Secreted DKK1 in the culture media was determined by using the human DKK1 ELISA kit (DKK100, R&D, Minneapolis, MN, USA) and calcium influx in osteoblasts was determined by using the Fura-2 Calcium Flux Assay kit (ab176766, Abcam, Cambridge, UK), according to the manufacturer’s protocols.

### 2.6. Immunoblotting (IB) and mRNA Analysis

For immunoblotting, the stimulated cells were washed with 1X PBS and lysed by 1X RIPA buffer containing proteinase and phosphatase inhibitors. The lysates were separated by SDS-PAGE, transferred to nitrocellulose membranes, immunoblotted with corresponding primary and secondary antibodies, and visualized with ECL detection kits (34580, Thermo Fisher, Waltham, MA, USA). Primary and secondary antibodies used for immunoblotting are described in Table 1. For mRNA analysis, RNA was extracted by Trizol reagent (15596018, Invitrogen, Carlsbad, CA, USA). 1 μg of total RNA was subjected to reverse transcription (K1622, Thermo Fisher, Waltham, MA, USA). qPCR was performed on CFX96 Real-time PCE detection system (Bio-Rad Laboratories, Hercules, CA, USA). The primers used for qPCR are described in Table 2. The expression of each target gene was normalized to GAPDH. Normalized expression values were averaged, and then average fold changes were calculated.

### 2.7. Immunofluorescence (IF)

Cultured or stimulated osteoblasts were washed two times with 1X PBS and fixed with 10% formalin for 15 min, followed by permeabilization with 1X PBS containing 0.1% Triton X-100 and 1% BSA in for 1 h, incubation with primary antibody overnight, washing with 1X PBS, and incubation with Cy3 or Alexa 488-conjugated secondary antibody for 1 h. The stained cells were washed with distilled water and mounted with DAPI (H1200, Vector, Burlingame, CA, USA). To visualize stained cells, immunofluorescence images were collected with a confocal microscope. Secondary antibodies used for immunofluorescence are described in Table 1.

### 2.8. Luciferase Assay

Approximately 1 kb of the human DKK1 promoter (positions +111 to −935) was used, as described previously [33]. SaOS2 cells were co-transfected with DKK1 promoter (1 μg/well) and Renilla (0.5 μg/well) under growth medium. Two days after transfection, the cells were lysed and luciferase activity was determined using the Luciferase assay (E1500, Promega, Madison, WI, USA) according to the manufacturer′s protocol. The luciferase activity was measured with a Luminometer (Berthold, Bad Wildbad, Land Baden-Württemberg, Germany) and normalized based on Renilla luciferase.

### 2.9. Trichloroacetic Acid (TCA) Precipitation

Methods for TCA precipitation were previously reported [38]. Briefly, osteoblasts were seeded at about 90% to 100% confluency. The next day, the growth media were replaced with serum-free DMEM media including 20 nM 1,25D3 or vehicle for a day. The cell supernatants were collected and subjected to TCA precipitation.

### 2.10. Chromatin Immunoprecipitation (ChIP) Assay

ChIP assays were prepared using the Chromatin Immunoprecipitation Assay Kit (17-295, Millipore, Burlington, MA, USA) according to the instructions. Briefly, 1,25D3-stimulated osteoblasts were cross-linked with 1% formalin for 15 min at room temperature and washed three times with pre-chilled 1× PBS containing protease inhibitors. The cells were collected by scraping and centrifugation and lysed in 1× SDS lysis buffer, followed by vortexing then sonication with Bioruptos (Cosmo Bio, Tokyo, Japan). The average length of DNA fragments ranged between 500 and 1000 bp. The lysates were then clarified by centrifugation and precleared using protein G-Sepharose beads for 1 h at 4 °C. The samples were then immunoprecipitated with 5 μg anti-C/EBPβ overnight and the immune complexes were washed with low-salt buffer, high-salt buffer, LiCl buffer, and TE buffer, and then eluted with elution buffer. Immunoprecipitated DNA was reverse cross-linked at 65 °C overnight and purified using a Genomic DNA purification kit (118-050, GeneAll, Seoul, Korea). A total of 1–2.5 ng of the purified DNA were subjected to qPCR using the specific primer for C/EBPβ binding element present in the DKK1 promoter.

### 2.11. Statistical Analysis

GraphPad Prism version 6 (GraphPad, San Diego, CA, USA) was used to analyze and present the data. All data were analyzed by analysis of variance, followed by an unpaired or paired *t*-test. Values are given as means ± standard deviations (SD). 

## 3. Results

### 3.1. 1,25D3 Transiently Increases Expression of DKK1 and C/EBPβ during Osteoblast Differentiation

**Figure 1:**
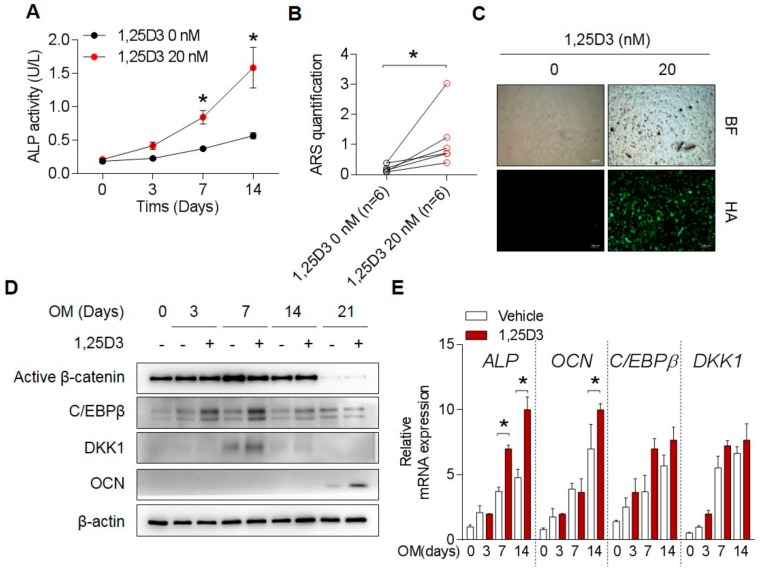
1,25D3 transiently increases expression of DKK1 and C/EBPβ during osteoblast differentiation. Osteoprogenitors were treated with 0 or 20 nM 1,25D3 under osteoblast differentiation. (**A**) Intercellular ALP activity at indicated days, (**B**) ARS quantification at 14 days, (**C**) Hydroxyapatite staining at 14 days. BF, Bright Field; HA, Hydroxyapatite. Representative data are shown. * *p* < 0.05; (mean ± SD; *n* = 6) (**D**) Protein levels were determined by immunoblotting at indicated days. (**E**) mRNA levels were determined by qPCR at indicated days. Representative data are shown. * *p* < 0.05; (mean ± SD; *n* = 3).

Consistent with previous reports, treatment with 1,25D3 in osteoprogenitors enhanced osteoblast differentiation indicators, such as ALP activity and staining (Figure 1A and Figure S1a), calcium deposition B and Figure S1b), hydroxyapatite formation (Figure 1C), and osteoblast-related promoters ALP, BSP, OSE, and OCN (data not shown) as markers for bone-forming activity. Treatment with 1,25D3 markedly increased C/EBPβ and DKK1 at day 7 and promoted the expression of OCN at day 21 (Figure 1D). mRNA levels of C/EBPβ, DKK1, and osteoblast differentiation-related ALP and OCN genes were increased as well (Figure 1E). These data suggest that 1,25D3 treatment in osteoblast differentiation led to the increase in C/EBPβ and DKK1 expression levels accompanied by enhanced differentiation status.

### 3.2. 1,25D3 Induces Expression of DKK1 in Osteoblasts through C/EBPβ

**Figure 2:**
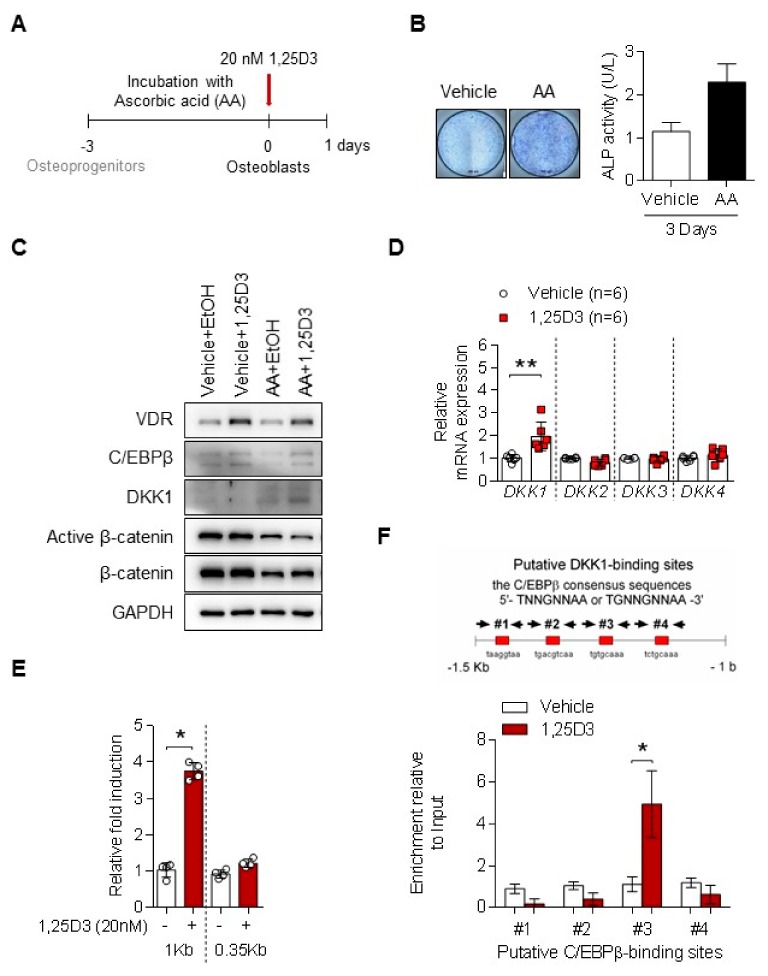
1,25D3 induces expression of DKK1 in osteoblasts through C/EBPβ. (**A**) Experimental scheme for induction of osteoblasts. (**B**) Osteoprogenitors were stimulated with 50 nM AA for 3 days, incubated with 20 nM 1,25D3 for a day, and analyzed by ALP staining and intracellular ALP activity. (**C**) Osteoprogenitors (vehicle) or osteoblasts (AA) were treated with 20 nM 1,25D3 for 24 h and analyzed by immunoblotting (*n* = 6). (**D**) DKK genes in 1,25D3-induced osteoblasts were analyzed by qPCR (*n* = 6). (**E**) Osteoblasts were transfected with 1 kb DKK1 or 0.35 kb DKK1 for 48 h, treated with 20 nM 1,25D3 for 24 h, and then analyzed with luciferase assay (*n* = 4). (**F**) Osteoblasts were treated with 20 nM 1,25D3 for 24 h then subjected to ChIP assay (*n* = 4). Representative data are shown. * *p* < 0.05, ** *p* < 0.01; (mean ± SD).

To establish the osteoblasts from osteoprogenitors, we treated with AA for 3 days (Figure 2A). Stimulation with AA increased ALP activity (Figure 2B) and RUNX2, an osteoblast marker in osteoprogenitors (hereafter as to osteoblasts), but the expression levels of C/EBPβ, COL1A1, OPN, and OCN genes were not statistically changed (Figure S2). Interestingly, 1,25D3 increased DKK1 expression in osteoblasts, but not in osteoprogenitors (Figure 2C). This effect depends on the 1,25D3 dose (Figure S1c). As shown in Figure 2D, stimulation with 1,25D3 upregulated mRNA levels of DKK1 in osteoblasts, but not of other DKK genes. Moreover, 1,25D3 treatment upregulated human DKK1 promoter activity, which includes the 1-kb region (from –935 to +111) (Figure 2E). DKK1 has four putative C/EBPβ-binding sites within human DKK1 promoter 1.5-kb region and a possible binding site, which is considered to be primarily a mechanism by which 1,25D3-stimulated C/EBPβ protein directly induces DKK1 transcript. We further showed that C/EBPβ protein by 1,25D3 stimulation binds #3 region of DKK1 promoter in osteoblasts, as revealed by ChIP (Figure 2F). These results suggest that C/EPBβ plays a critical role in DKK1 expression of 1,25D3-induced osteoblasts.

### 3.3. C/EBPβ Regulates 1,25D3-Induced DKK1 Expression

**Figure 3:**
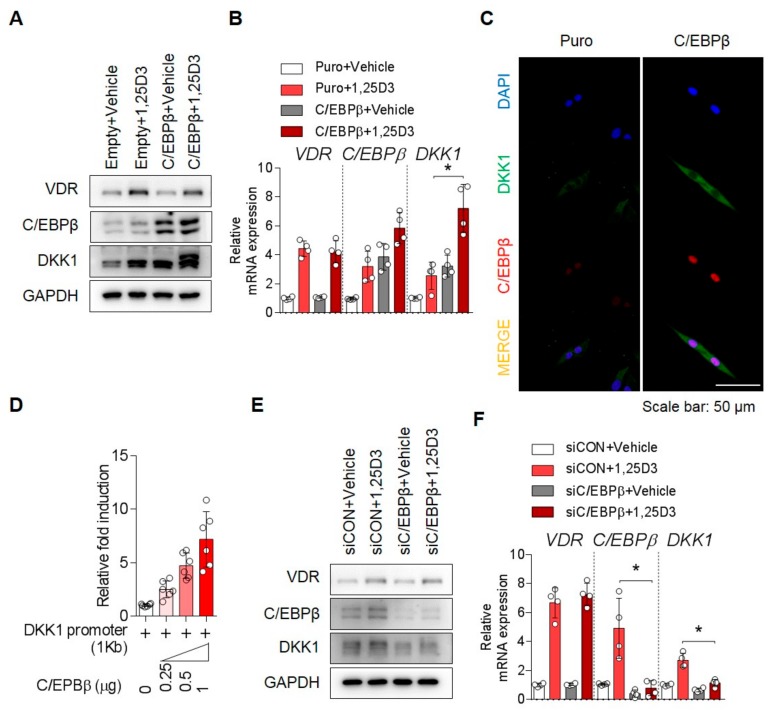
C/EBPβ regulates 1,25D3-induced DKK1 expression. Osteoblasts were transfected with empty vector or C/EBPβ, incubated for 48 h, and stimulated with 1,25D3 for 24 h. Analysis of (**A**) immunoblotting for protein level and (**B**) qPCR for mRNA level (*n* = 4). (**C**) Osteoblasts were transfected with empty vector or C/EBPβ for 48 h and transfected cells were stained with anti-DKK1 (green), anti-C/EBPβ (red), and DAPI (blue). Scale bar is 50 μm. (**D**) Osteoblasts were co-transfected with 1 kb DKK1 promoter and C/EBPβ in a dose-dependent manner for 48 h and analyzed by luciferase assay (*n* = 6). Osteoblasts were transfected with siRNA against control (CON) or C/EBPβ, incubated for 48 h, and stimulated with 1,25D3 for 24 h. Analysis of (**E**) immunoblotting for protein level and (**F**) qPCR for mRNA level (*n* = 4). Representative data are shown. * *p* < 0.05; (mean ± SD).

To determine whether C/EBPβ is required for 1,25D3-induced DKK1 expression, the expression of DKK1 was examined by manipulation of the C/EBPβ gene in the presence or absence of 1,25D3. We manipulated expression of the C/EBPβ gene or its controls for 48 h in osteoblasts and then treated with 1,25D3 for 24 h. As shown in Figure 3, stimulation with 1,25D3 enhanced both mRNA and protein expression of DKK1 in the cells overexpressing the C/EBPβ gene (Figure 3A–D and Figure S3a). On the contrary, 1,25D3-induced upregulation of C/EBPβ and DKK1 was inhibited by C/EBPβ knockdown using siRNA (Figure 3E,F). Thus, our data implicate the importance of the C/EPBβ gene in 1,25D3-mediated DKK1 expression.

### 3.4. 1,25D3 Stimulates Secretion of DKK1 Protein in Osteoblasts.

**Figure 4:**
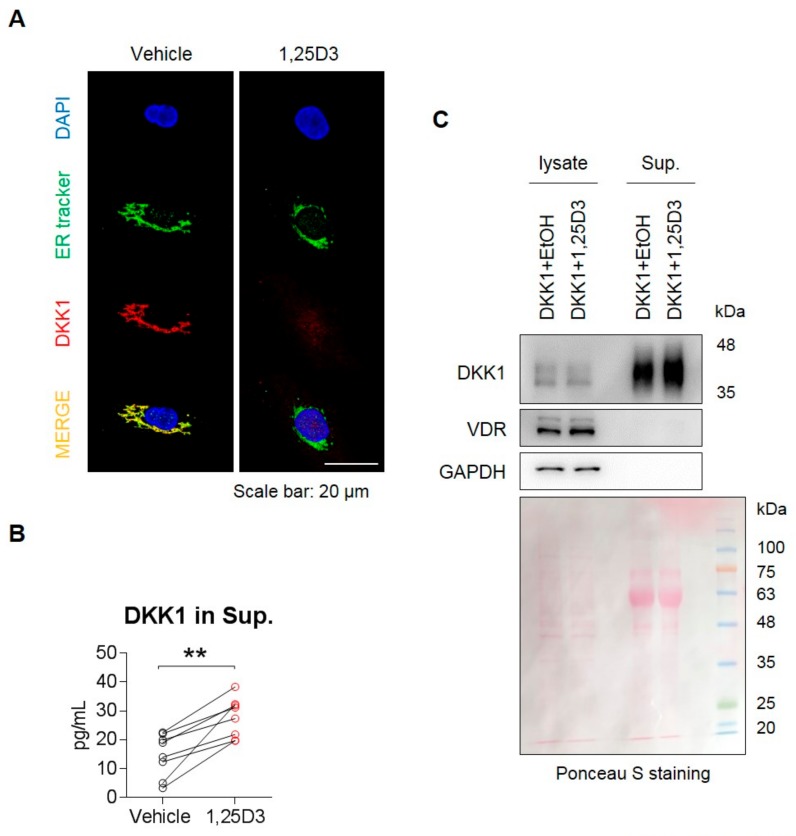
1,25D3 stimulates secretion of DKK1 protein in osteoblasts. (**A**) Osteoblasts were treated with 20 nM 1,25D3 for 24 h and stained with ER tracker (green), anti-DKK1 (red), and DAPI (blue). Scale bar is 20 μm. Representative data are shown (*n* = 3). (**B**) Osteoblasts were treated with 20 nM 1,25D3 for 24 h, then the culture supernatant was collected and secreted DKK1 protein was measured in the supernatant using ELISA. ** *p* < 0.01 (mean ± SD; *n* = 6). (**C**) Osteoblasts were transfected with 2 μg DKK1 plasmid for 48 h, and then treated with 1,25D3 for 24 h. Proteins secreted in the cell supernatant by 1,25D3 stimulation were collected, precipitated by trichloroacetic acid (TCA) and detected by SDS-PAGE/immunoblotting. Ponceau S staining was used as culture supernatant controls. Representative data are shown (*n* = 3).

Given that DKK1 is a secretory protein, which is originally located within the ER of cells, we used an ER tracker to confirm the location of DKK1 in osteoblasts. Next, we examined the effect of 1,25D3 on secretion of the DKK1 protein in osteoblasts. DKK1 protein was not detected in the ER in 1,25D3-stimulated osteoblasts (Figure 4A), but instead appeared in the cell supernatant, indicating that DKK1 protein was secreted by 1,25D3 stimulation (Figure 4B). Furthermore, when stimulated with 1,25D3 in overexpressing DKK1 osteoblasts (Figure S3b), DKK1 protein was slightly reduced in 1,25D3-stimulated cells lysates, but an increase in secreted DKK1 protein was observed in cells supernatant of those (Figure 4C). Furthermore, we confirmed that 1,25D3 allows calcium entry into osteoblasts and induces ER stress in osteoblasts in a dose-dependent manner (Figure S4). These findings suggest that 1,25D3 stimulation is involved in the secretion of the DKK1 protein in osteoblasts.

### 3.5. DKK1 Blockade Inhibits Mineralization of Osteoblast Differentiation

**Figure 5:**
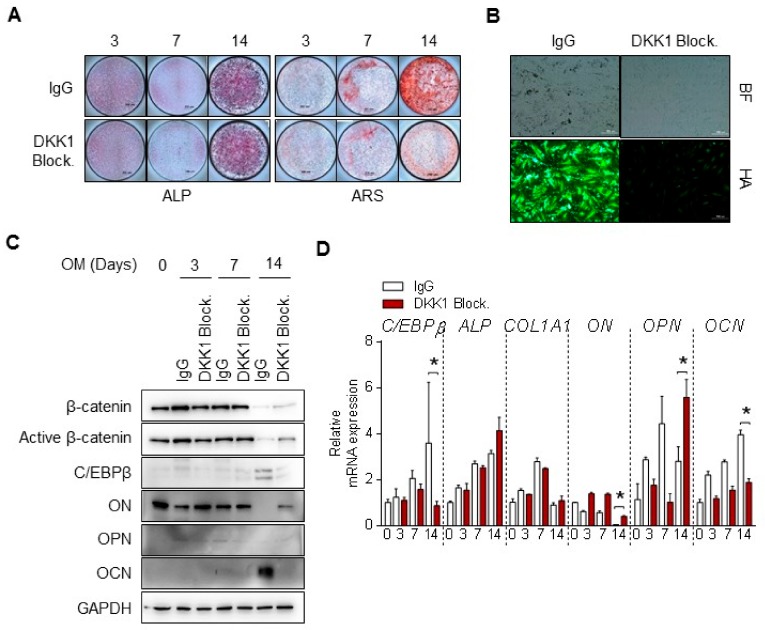
DKK1 blockade inhibits mineralization of osteoblast differentiation. Osteoblasts were treated with 1 μg anti-DKK1 or IgG as controls during osteoblast differentiation (*n* = 3). At indicated days, analysis of (**A**) results of ALP and ARS staining; scale bar is 200 μm, (**B**) hydroxyapatite staining; BF, Bright Field; HA, Hydroxyapatite; Scale bar is 500 μm, (**C**) immunoblotting for proteins, and (**D**) qPCR for mRNA. Representative data are shown (*n* = 3). * *p* < 0.05 (mean ± SD; *n* = 3).

To determine whether regulation of DKK1 affects osteoblast differentiation, we examined the treatment of DKK1 blockade during osteoblastic differentiation and observed changes in differentiation indicators. Intriguingly, blocking DKK1 in osteoblasts blocked mineralization such as calcium deposits and hydroxyapatite formation, but not ALP activity (Figure 5A,B). Moreover, OCN levels at 14 days were clearly decreased with DKK1 blockade compared to IgG (Figure 5C). mRNA levels of ALP and COL1, early markers of differentiation, did not change during osteoblast differentiation, but OCN expression was reduced with DKK1 blockade (Figure 5D). Conversely, cells with DKK1 overexpression or recombinant DKK1 protein exhibited slightly increased calcified nodule and hydroxyapatite formation (Figure S5). Taken together, these results suggest that DKK1 is required for the mineralization of osteoblasts.

## 4. Discussion

In this present study, we showed that 1,25D3 treatment for osteoblast differentiation led to enhancement of C/EBPβ and DKK1 expression as well as mineralization of osteoblasts. We found that 1,25D3 treatment in osteoblasts facilitated DKK1 expression, which in turn was directly regulated by the 1,25D3-responsible C/EBPβ gene. Overexpression of C/EBPβ significantly increased DKK1 expression, whereas a knockdown of C/EBPβ decreased DKK1 expression. The modulation of DKK1 expression by C/EBPβ was controlled in response to 1,25D3 treatment. 1,25D3 also stimulated secretion of DKK1 in osteoblasts via ER stress. Importantly, exposure to anti-DKK1 inhibited mineralization of osteoblast differentiation. These results strongly demonstrate that 1,25D3 induces DKK1 expression via C/EBPβ to promote the mineralization of osteoblast differentiation.

Increases in osteoblastic activity and bone formation upon 1,25D3 treatment in vivo and in vitro are evident [39,40]. These changes are accompanied by a striking increase in C/EBPβ and RUNX2 during osteoblast differentiation. The role of RUNX2 in governing multiple steps of osteoblast differentiation is well known, but relatively little attention has been paid to the role of C/EBPβ in osteoblast differentiation. As shown in Figure S6, 1,25D3 binds VDR to induce C/EBPβ, which regulates DKK1 expression. 1,25D3 affected DKK1 activation during osteoblast differentiation and blocked Wnt/β-catenin signaling, thereby inducing mineralization in osteoblasts. Thus, this study provides insight into the functional role DKK1 plays in response to 1,25D3 in the mineralization of osteoblasts, although 1,25D3 promotes osteoblast differentiation.

Osteoblast differentiation has three sequential steps: proliferation, matrix maturation, and mineralization. ALP is an important early indicator and marker for matrix maturation and the RUNX2 gene is the master regulator for osteoblast differentiation and bone formation. Stimulation of preosteoblast MC3T3-E1 cells with AA induced osteoblast features was previously reported [41,42]. Thus, we applied this concept and induced osteoblasts from osteoprogenitors with AA treatment for three days (Figure 2A). As shown in Figure 2B and Figure S2, AA treatment in osteoprogenitors resulted in the upregulation of both the ALP and RUNX2 genes. These results suggest a mechanism in which AA affects osteoprogenitor commitment to osteoblast differentiation through the induction of the ALP and RUNX2 genes. Given that mRNA expression and secretion of DKK1 were high at day 7 of osteoblast differentiation, we cultured osteoprogenitors in the presence of AA for 7 days, but this showed too much collagen synthesis on the cells. Rather, the DKK1 induction response to 1,25D3 in osteoblasts was more increased at day 3 of AA incubation compared to day 7. Thus, we optimized the experimental conditions so that 1,25D3 induced DKK1 expression in osteoblasts for 1 day following the osteoprogenitor treatment with AA for 3 days.

Previously, it was reported that DKK1 was expressed in osteoblasts and osteocytes [43]. However, the functional role of DKK1 in osteoblasts is not fully understood. With microarray data, we have shown that DKK1 expression was markedly increased during osteoblast differentiation, while DKK2, 3, and 4 were not increased (Figure S7a). Secretory and mRNA expression levels of DKK1 gradually increased up to day 7 and then decreased during osteoblast differentiation (Figure S7b,c). The microarray data were validated by mRNA and protein expressions of DKK1. Furthermore, four DKK genes have been identified in mammals, among which DKK1 and DKK2 have been well characterized and found to act as antagonists to the canonical Wnt pathway by binding to LRP5/6. In this study, we show that 1,25D3 induces DKK1 expression via C/EBPβ to promote mineralization of osteoblasts, demonstrating some regulatory effects and molecular mechanisms of DKK1 in osteoblasts. However, the mechanisms of how 1,25D3-induced DKK1 affects the suppression of Wnt/β-catenin signaling to regulate mineralization of osteoblasts remains to be clarified.

In vivo experiments have revealed that Dkk1 transgenic mice have decreased bone mass, whereas Dkk1-null mice osteoblasts have increased bone formation and mass [19,20,21]. By contrast, Dkk2-null mice exhibit a lack of mineralization and bone-forming features, and that Dkk2 not only expresses during osteoblast differentiation but stimulates the mineralization of primary osteoblasts [44]. According to the Dkk genes in mice, different characteristics of bone formation were observed in this study. We found that using an anti-DKK1 neutralizing agent in osteoblasts blocked mineralization such as calcium deposits and hydroxyapatite formation, but not ALP (Figure 5). Moreover, DKK1 overexpression or recombinant protein for osteoblast differentiation did not affect ALP staining but slightly exhibited calcified nodules, as revealed by ARS staining (Figure S5). These results are in line with the role found in the Dkk2-null mice study. Hence, it appears that DKK1 has a role in mineralization of human osteoblasts.

Since patients with multiple myeloma (MM) have shown high DKK1 levels that were correlated with osteolytic bone damage, an anti-DKK1 neutralizing agent has been developed and clinically used in MM [45,46]. Myeloma cells in MM patients extensively secrete DKK1 to inhibit proliferation of mesenchymal stem cells (MSC) or early-stage osteoprogenitors, leading to decreased bone formation at osteolytic bone. To understand the pathological role of DKK1 in osteoprogenitors, we designed an experiment wherein osteoprogenitors were not fully confluent in a well plate (about 50% to 70% cell density) and were stimulated with human recombinant DKK1 protein. As expected, treatment with DKK1 protein inhibited the proliferation rate of osteoprogenitors (data not shown). It seems likely that many granules such as senescent cells in DKK1 protein-treated osteoprogenitors were observed. These results imply that DKK1 plays a negative role in the proliferation of osteoprogenitors.

In general, secretory proteins are known to be regulated by ER stress [47]. We showed that 1,25D3 treatment caused secretion of DKK1 protein and induction of ER stress markers (Figure 4 and Figure S4a). Moreover, 1,25D3 triggered calcium influx in cells (Figure S4b). Therefore, further studies will be focused on physiological function or identifying the mechanisms by which induction of calcium-ER stress by 1,25D3 regulates secretion of DKK1 in osteoblasts.

In summary, we have suggested a molecular mechanism whereby 1,25D3 induces DKK1 expression via C/EBPβ to promote the mineralization of osteoblast differentiation.

## Figures and Tables

**Table 1:** Primary and secondary antibodies used in IB, IF, ChIP.

Antigen	Manufacturer	Species, Type	Catalog Number	Dilution
DKK1	Santa Cruz/TX, USA	Mouse monoclonal	sc-374574	1:1000
DKK1	Cell Signaling/MA, USA	Rabbit monoclonal	48367	1:1000
C/EBPβ	Santa Cruz/TX, USA	Mouse monoclonal	sc-7962	1:1000
VDR	Cell Signaling/MA, USA	Rabbit monoclonal	12550	1:1000
Active β-catenin	Cell Signaling/MA, USA	Rabbit monoclonal	19807	1:1000
β-catenin	Cell Signaling/MA, USA	Rabbit polyclonal	9562	1:1000
RUNX2	Cell Signaling/MA, USA	Rabbit monoclonal	12556	1:1000
RUNX2	Santa Cruz/TX, USA	Mouse monoclonal	sc-101145	1:1000
Osteonectin, ON	Santa Cruz/TX, USA	Mouse monoclonal	sc-73472	1:1000
Osteopontin, OPN	R&D System/MN, USA	Goat polyclonal	AF1433	1:2000
Osteocalcin, OCN	Santa Cruz/TX, USA	Mouse monoclonal	sc-365797	1:1000
Osteocalcin, OCN	Abcam/Cambridge, UK	Mouse monoclonal	ab13420	1:1000
IgG	Millipore/MA, USA	Rabbit monoclonal	pp64	1:10000
β-actin	Cell Signaling/MA, USA	Rabbit monoclonal	4970	1:10000
β-actin	Cell Signaling/MA, USA	Mouse monoclonal	3700	1:10000
GAPDH	Cell Signaling/MA, USA	Rabbit monoclonal	2118	1:10000
Alexa-488	ThermoFisher/MA, USA	Mouse	A11001	1:100
Cy3	ThermoFisher/MA, USA	Rabbit	A10520	1:100
HRP	Jackson ImmunoResearch/CA, USA	Mouse	115-035-003	1:2000
HRP	Jackson ImmunoResearch/CA, USA	Rabbit	111-035-003	1:2000

**Table 2:** Primer sequences for qPCR and ChIP assay and siRNA target sequences.

Gene	5′-Forward-3′	5′-Reverse-3′
DKK1 (mRNA)	CACACCAAAGGACAAGAAGG	CAAGACAGACCTTCTCCACA
C/EBPβ (mRNA)	CGACGAGTACAAGATCCGGC	TGCTTGAACAAGTTCCGCAG
VDR (mRNA)	TGGAGACTTTGACCGGAACG	GGGCAGGTGAATAGTGCCTT
ALP (mRNA)	ACGAGCTGAACAGGAACAACGT	CACCAGCAAGAAGAAGCCTTTG
RUNX2 (mRNA)	GTGGCCTTCAAGGTGGTAG	ACTCTTGCCTCGTCCACTC
COL1A1 (mRNA)	AGTGGTTTGGATGGTGCCAA	GCACCATCATTTCCACGAGC
ON (mRNA)	GGATGAGAACAACACCCCCA	TTTGCAAGGCCCGATGTAGT
OPN (mRNA)	AGCAGCTTTACAACAAATACCCAG	TTACTTGGAAGGGTCTGTGGG
OCN (mRNA)	AGCCACCGAGACACCATGAGA	CTCCTGAAAGCCGATGTGGTC
DKK2 (mRNA)	GAGGTATTGCCACAGTCCCC	GATGCCATTATTGCAGCGGG
DKK3 (mRNA)	TATGTGTGCAAGCCGACCTT	CTCCTCCATGAAGCTGCCAA
DKK4 (mRNA)	CTGTGCTACATGTCGTGGGT	TCCTTCTGCATGTGTGCCAT
GAPDH (mRNA)	CAAGATCATCAGCAATGCC	CTGTGGTCATGAGTCCTTCC
DKK1 promoter 1 (ChIP)	TTTGTATTCACTGTGCCCCTCC	CCTAGAGCCCTGGCATTGG
DKK1 promoter 2 (ChIP)	TCCACACACCAATTTCAATGACG	GGGACCACGCAATACCCTTT
DKK1 promoter 3 (ChIP)	TCTAAACGCCAGTCTCTCGC	CGGCTTTGAGGTCCTTCAGT
DKK1 promoter 4 (ChIP)	ACCTCAAAGCCGGGGATCTA	TTGCCCCTCTCCTTTATGCC
**siRNA**	**5′-Sense-3′**	**5′-Antisense-3′**
siControl (siCON)	CCUCGUGCCGUUCCAUCAGGUAGUU	CUACCUGAUGGAACGGCACGAGGUU
siC/EBPβ	ACAACAUCGCCGUGCGCAAUU	UUGCGCACGGCGAUGUUGUUU
siVDR	GGAGUUCAUUCUGACAGAUUU	AUCUGUCAGAAUGAACUCCUU

## Supplementary Materials

The following are available online at https://www.mdpi.com/2073-4409/9/1/236/s1, Figure S1: 1,25D3 dose dependently enhances osteoblast differentiation of osteoprogenitors, Figure S2: Ascorbic acid (AA) induces ALP and RUNX2 expressions in osteoprogenitors, Figure S3: Results of semi-quantitative RT-PCR on Figure 3D and Figure 4C, Figure S4: 1,25D3 stimulates calcium influx and ER stress in osteoblasts, Figure S5: DKK1 promotes mineralization of osteoblasts, Figure S6: VDR regulates 1,25D3-induced C/EBPβ expression, Figure S7: DKK1 is increased during osteoblast differentiation.
