# Supplementary material for: DKK1 Induced by 1,25D3 Is Required for the Mineralization of Osteoblasts
Source: Cells. 2020 Jan 17;9(1):236. doi: 10.3390/cells9010236 (PMC7017072; doi:10.3390/cells9010236)
Supplement: Supplementary file 1 [file cells-09-00236-s001.pdf]

SUPPLMENTARY DATA

DKK1 induced by 1,25D3 is required for the mineralization of osteoblast

Figure S1. 1,25D3 dose dependently enhances osteoblasts differentiation of osteoprogenitors.

Figure S2. Ascorbic acid (AA) induces ALP and RUNX2 expressions in osteoprogenitors.

Figure S3. Results of semi-quantitative RT-PCR on Figure 3D and Figure 4C.

Figure S4. 1,25D3 stimulates calcium influx and ER stress in osteoblasts.

Figure S5. DKK1 promotes mineralization of osteoblasts.

Figure S6. VDR regulates 1,25D3-induced C/EBPβ expression

Figure S7. DKK1 is increased during osteoblasts differentiation

Supplementary data

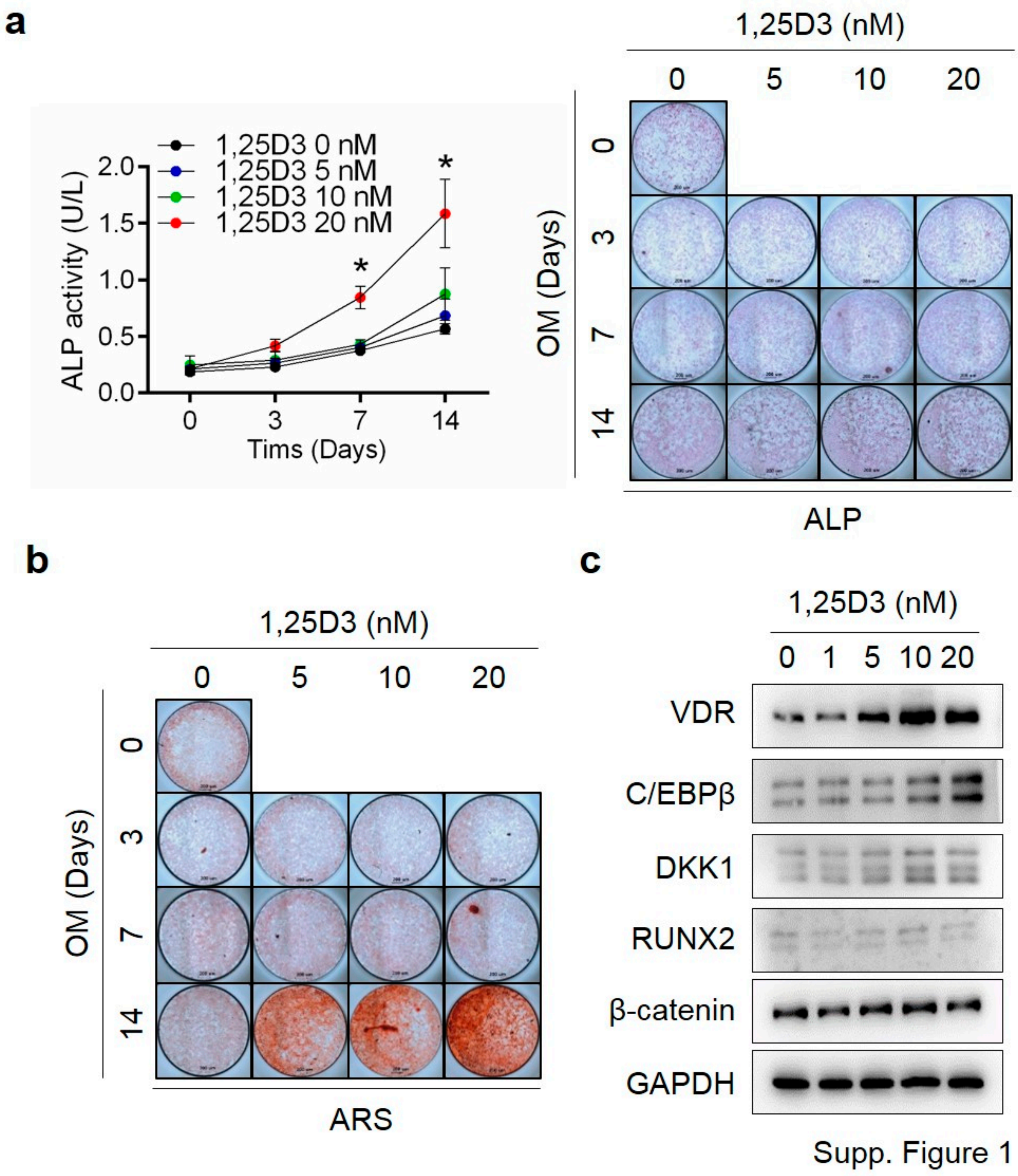

Figure S1. 1,25D3 dose dependently enhances osteoblasts differentiation of osteoprogenitors. Osteoprogenitors were treated with different 1,25D3 dose during osteoblasts differentiation. Osteogenic medium was changed every 3 days. As indicated day, the differentiated cells were subjected to (a) ALP and ARS staining and (b) Intercellular ALP activity. (c) Osteoblasts were

treated with different dose of 1,25D3 for a day and analyzed by immunoblotting. Independent experiments were performed four times. Representative images are presented, scale bar 200μm.

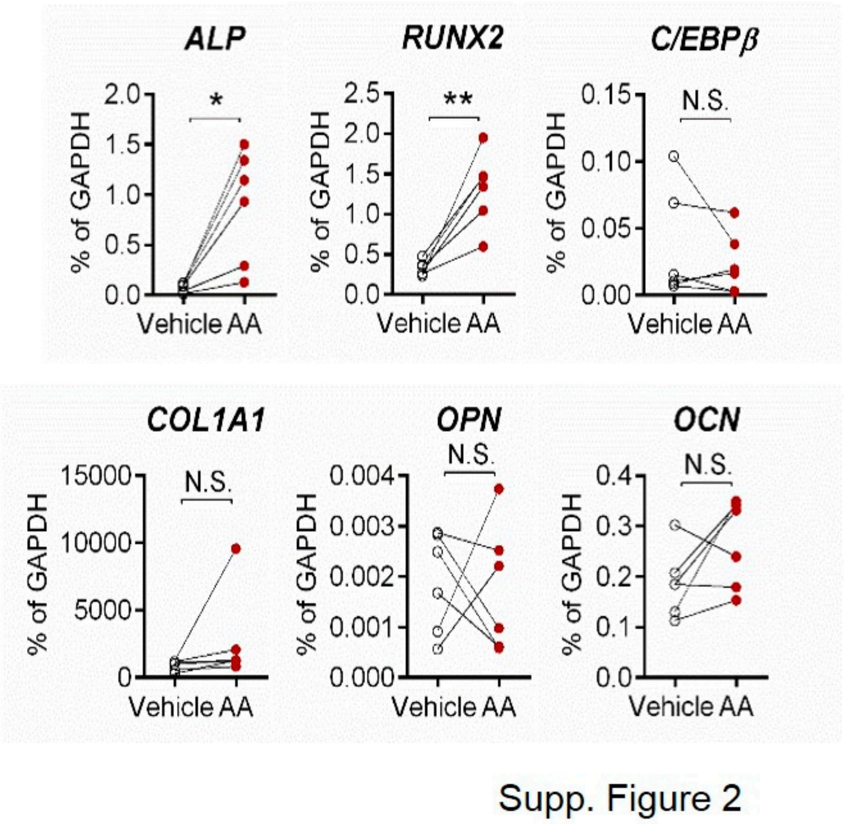

Figure S2. Arscobic actid (AA) induces ALP and RUNX2 expressions in osteoprogenitors. Osteoprogenitors were stimulated with 50 nM AA for 3 days and incubated with 20 nM 1,25D3 for a day. mRNA results of ALP, RUNX2, C/EBPβ, COL1A1, OPN, and OCN were analyzed by qPCR (n=6).

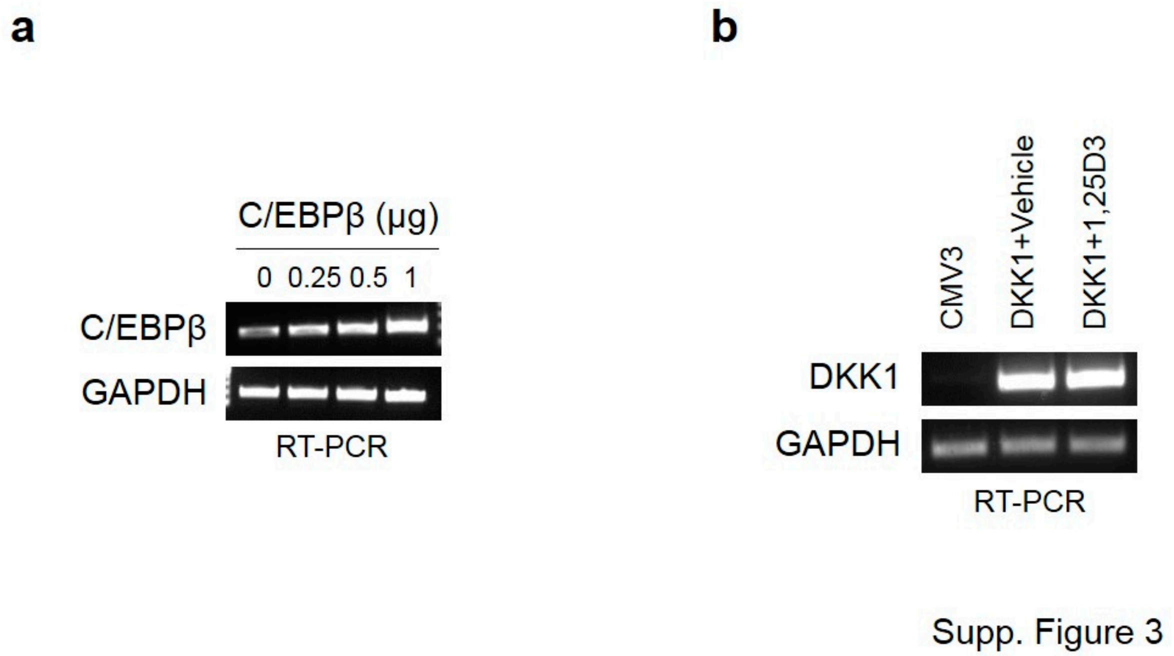

Figure S3. Results of semi-quantitative RT-PCR on Figure 3D and Figure 4C.

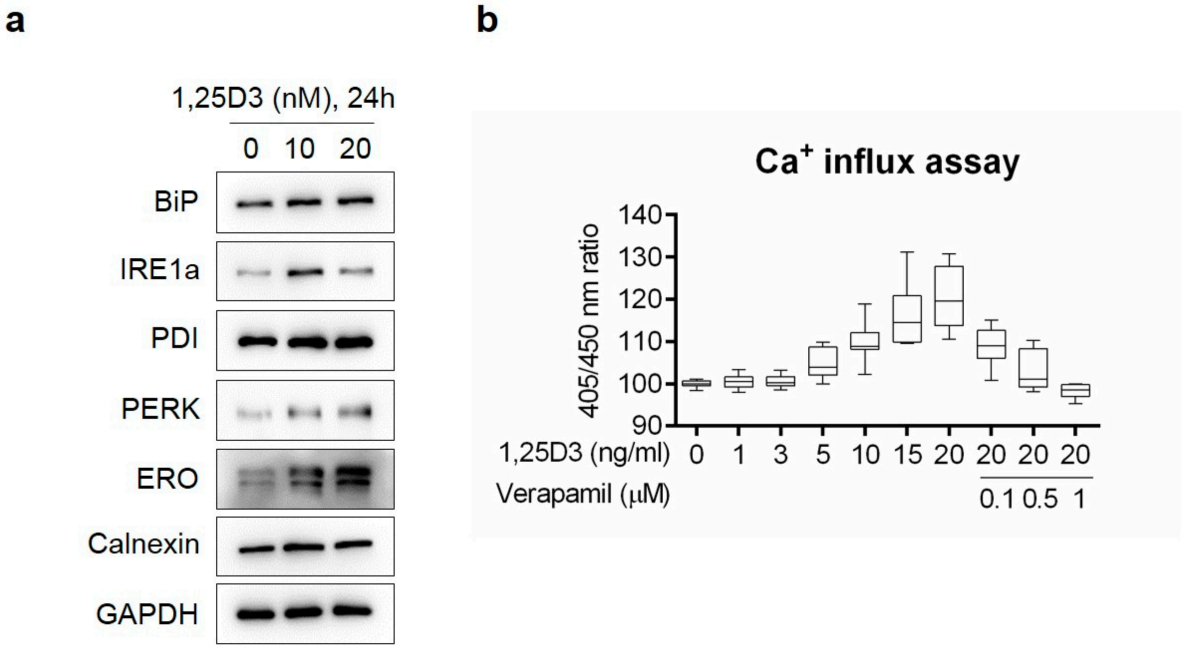

Supp. Figure 4

Figure S4. 1,25D3 stimulates calcium influx and ER stress in osteoblasts. (a) Osteoblasts were treated with different dose of 1,25D3 in absence or presence of Verapamil as calcium channel blocker. Osteoblasts were treated with 1,25D3 as indicated dose for 24h and analyzed by immunoblotting (n=2). (b) Calcium influx in osteoblasts were determined by Fura-2 kit (n=3). Data are presented as means ± SDs.

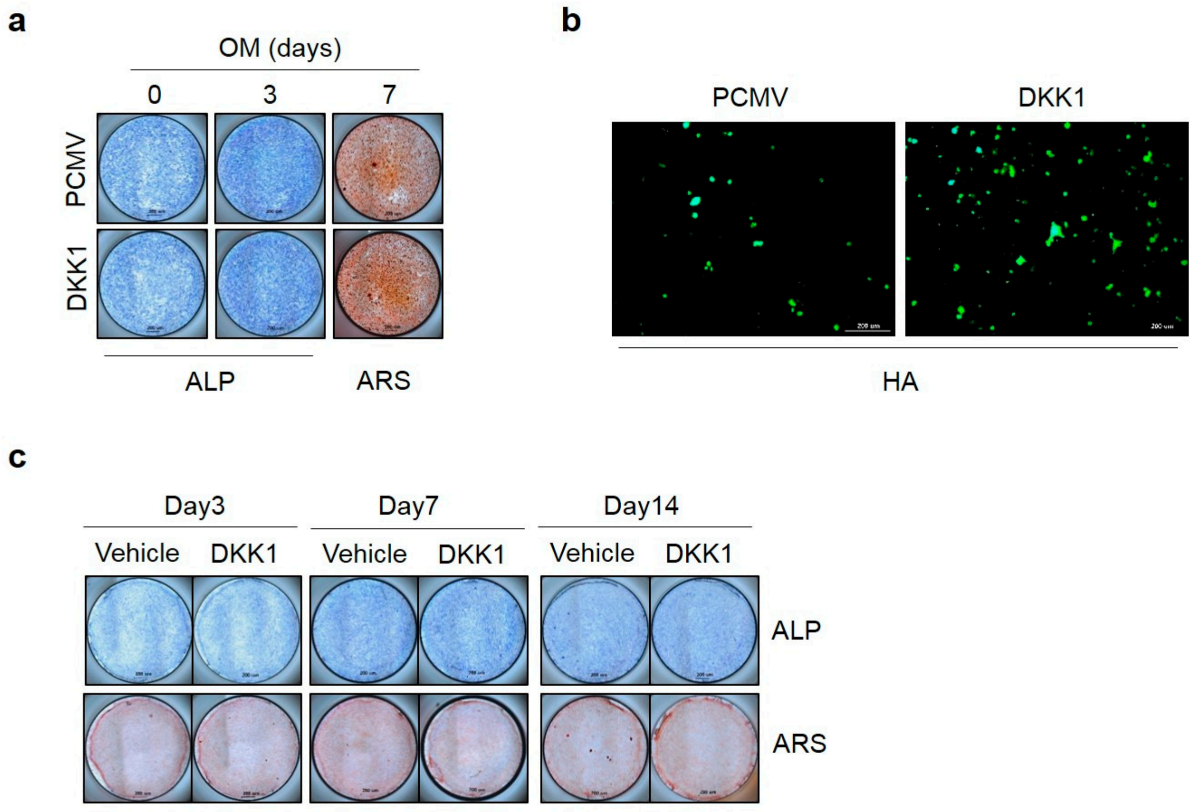

Supp. Figure 5

Figure S5. DKK1 promotes mineralization of osteoblasts. SaOS2 cells were transfected with empty vector or DKK1 plasmid and then induced with osteoblasts differentiation. Results of (a) ALP and ARS staining and (b) Hydroxyapatites staining. (c) Osteoblasts were treated with recombinant DKK1 protein or Vehicle as controls during osteoblast differentiation. At indicated days, the differentiated cells were analyzed by ALP or ARS staining. Representative images are shown. Scale bar is 200 µm.

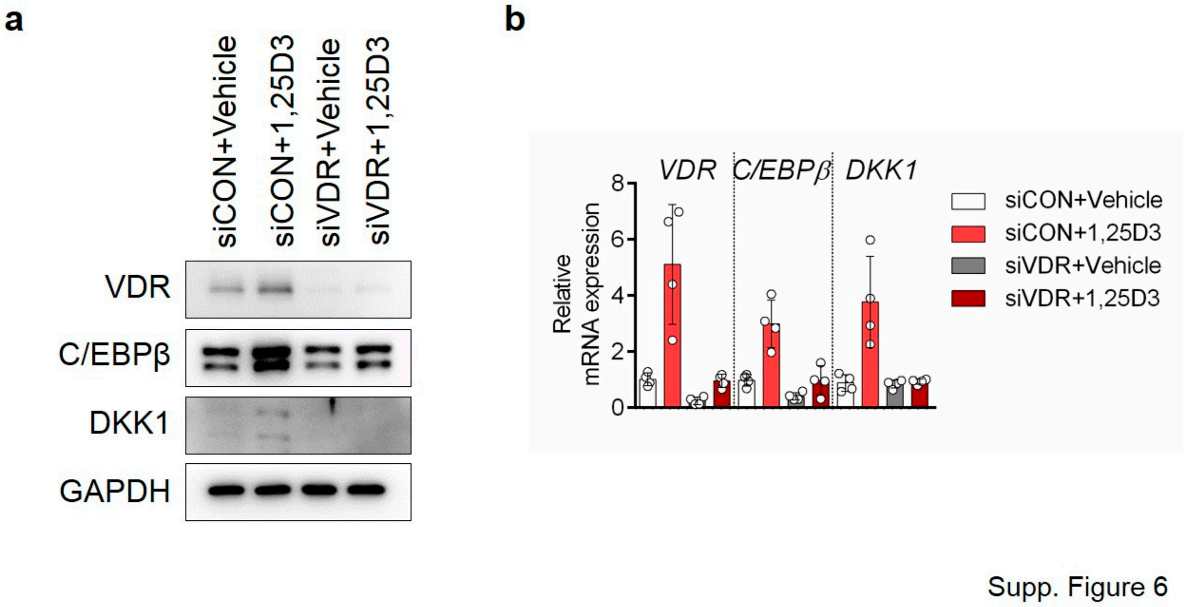

Figure S6. VDR regulates 1,25D3-induced C/EBPβ expression. Osteoblasts were transfected with siRNA against VDR or control (CON), incubated for 48h, and stimulated with 1,25D3 for 24h. Analysis of (a) Immunoblotting for protein level and (b) qPCR for mRNA level (n=4).

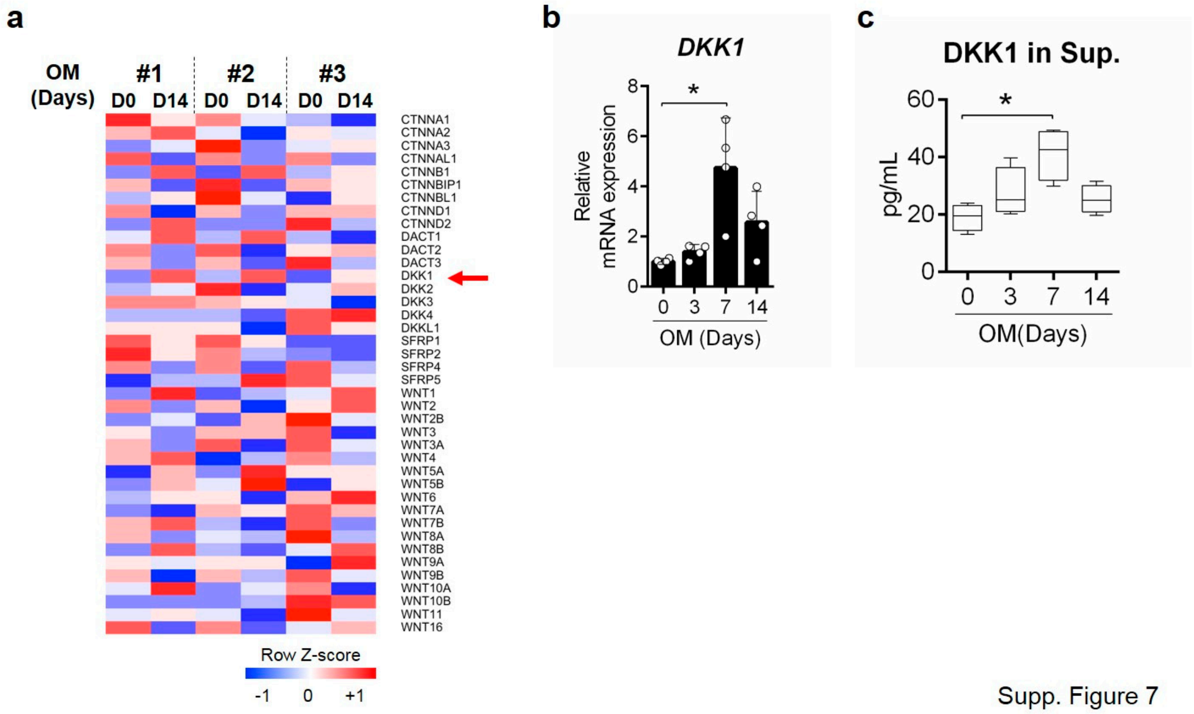

Figure S7. DKK1 is increased during osteoblasts differentiation. (a) Osteoprogenitors were differentiate into osteoblasts for 14 days and performed using microarray. The genes corresponding to the Wnt signaling are expressed in the heat map. Blue, low expression; red, high expression. Osteoprogenitors were induced to differentiate with osteoinductive agents as indicated days and analyzed by (b) qPCR for mRNA level of DKK1 and (c) ELISA for secreted DKK1 protein in cells supernatant.
